# Advances in Molecular and Translational Medicine: 2nd Edition

**DOI:** 10.3390/ijms26199633

**Published:** 2025-10-02

**Authors:** Mariarosaria Boccellino

**Affiliations:** Department Life Sciences, Health and Health Professions, Link University, 00165 Rome, Italy; m.boccellino@unilink.it

Translational medicine is a dynamic and evolving discipline that bridges scientific discoveries and clinical practice to deliver effective healthcare interventions [1,2,3]. By integrating molecular research with patient-centered clinical practice, this field aims to shorten the path from laboratory findings to therapeutic solutions, ultimately enhancing the quality of healthcare [4,5]. Through the investigation of disease mechanisms at the cellular and molecular levels, translational medicine promotes the development of targeted, personalized therapies [6,7]. This progress is driven by strong collaboration among researchers, clinicians, and healthcare providers, fostering innovation and a deeper understanding of human disease [8,9]. Following the success of the first edition, the second edition of the Special Issue *Advances in Molecular and Translational Medicine* brought together researchers and clinicians from around the world who shared their latest findings in the field, with a particular emphasis on clinical applicability and patient-centered outcomes. The contributions included novel molecular discoveries, innovative preclinical models, biomarker identification, and clinical studies. Collectively, these findings advanced our understanding of disease mechanisms and supported the development of targeted, patient-centered therapies. This Special Issue provides an excellent collection of recent research and review articles that explore emerging molecular mechanisms, diagnostic biomarkers, and therapeutic strategies across a spectrum of inflammatory, immune-mediated, and degenerative diseases. Building on the themes of precision medicine and translational research, this collection emphasizes the growing role of omics technologies, immune checkpoint regulation, metabolic pathways, and drug repurposing in understanding disease pathophysiology and guiding targeted interventions. Particularly noteworthy are the insights into how systemic inflammation, immune dysregulation, and cellular stress responses intersect in conditions such as organ transplantation, neurodegeneration, and chronic inflammation. Together, these contributions reflect the ongoing shift toward integrative and mechanism-based approaches in biomedical research and clinical management.

Łyżwa et al. [10] explored whether component-specific IgE testing could replace the oral food challenge (OFC) in diagnosing peanut allergy in sensitized children. The authors analyzed a cohort of 74 peanut-sensitized children, comparing their specific IgE responses to major peanut components (Ara h 1, 2, 3, and 6) with the clinical outcomes of OFCs. The results showed that component-resolved diagnostics (CRDs) were able to distinguish allergic from tolerant individuals with a certain degree of accuracy. However, component-specific IgE levels did not reliably predict either the severity of allergic reactions or the threshold dose required to elicit symptoms. Notably, in children with more severe reactions, CRD results were insufficient to replace OFC. The study suggests that while CRDs represent a valuable screening tool, particularly for identifying lower-risk patients, these cannot yet substitute the oral food challenge as the definitive diagnostic method. Further research is needed to optimize the clinical application of CRDs and better define their limitations and potential.

Zalewski et al. [11] investigated the dysregulation of key angiogenesis- and inflammation-related regulators in patients with varicose veins. The study involved 40 individuals with varicose veins and 24 healthy controls, analyzing the expression of 18 related genes in peripheral blood mononuclear cells (PBMCs) via real-time PCR, and measuring the levels of six proteins in plasma using ELISA. Compared to controls, patients exhibited significantly elevated PBMC expression of *CCL5*, *PDGFA*, and *VEGFC*, along with higher plasma levels of TGF-α, TGF-β1, and VEGF-A. Conversely, PBMC expression of *VEGFB* and plasma levels of VEGF-C were significantly reduced. No correlation was observed between these molecular alterations and the anatomical localization of the varicose veins. These findings suggest that disrupted VEGF and TGF signaling, along with chemokine-mediated inflammatory pathways, may play a role in varicose vein pathogenesis. Elevated CCL5 may promote inflammatory recruitment via CCR receptors, while VEGF-A and PDGFA likely contribute to aberrant angiogenesis and vascular remodeling in chronic venous disease. Although limited by its focus on selected regulators and by the absence of direct vascular tissue analysis, the study proposes a set of circulatory biomarkers with potential relevance for early detection or monitoring of varicose veins. Further research is needed to validate these markers and clarify whether these dysregulations reflect causative mechanisms or secondary disease features.

In a novel approach to addressing the systemic impact of oral pathogens, Chen et al. [12] evaluated the protective role of the hTERT-derived peptide GV1001 against *Porphyromonas gingivalis*-induced periodontitis and its associated complications in ApoE-deficient mice. In this experimental model, repeated gingival inoculations with *P. gingivalis* induced periodontitis, atherosclerosis, and Alzheimer’s disease-like pathologies. Treatment with GV1001 significantly reduced local and systemic inflammation, including alveolar bone loss, vascular lipid deposition, endothelial-to-mesenchymal transition (EndMT), and foam cell formation. Notably, GV1001 also inhibited the expression of Cluster of Differentiation 47 (CD47) on arterial smooth muscle cells, a molecule implicated in vascular inflammation, impaired phagocytosis, and aberrant vascular remodeling in chronic disease. Furthermore, GV1001 inhibited the accumulation of Alzheimer’s-related biomarkers (Aβ42 and p-Tau) in the brain, and prevented the aggregation of *P. gingivalis* DNA, lipopolysaccharide (LPS), and gingipains in gingival, vascular, and neural tissues. These findings position GV1001 as a promising multifunctional preventive agent. Beyond its established anti-inflammatory, antioxidant and anti-osteoclastogenic effects, the downregulation of CD47 suggests a novel mechanism by which GV1001 attenuates vascular dysfunction and promote resolution of inflammation. The work suggests potential for targeting oral–systemic links in chronic inflammatory and cardio-neurodegenerative diseases, particularly in genetically predisposed contexts.

Lim et al. [13] conducted a prospective, randomized, controlled, multicenter pilot study to assess the utility of an integrated risk score based on omics-derived biomarkers for predicting acute rejection (AR) in high-immunologic-risk kidney transplant recipients (KTRs). The study enrolled 40 patients, monitoring five key biomarkers: blood mRNA (three-gene signature), urinary exosomal miRNA (three-gene signature), urinary mRNA (six-gene signature), and two urinary exosomal proteins (hemopexin and tetraspanin-1), collected at baseline and throughout the first year post transplant. An integrated risk score was calculated and used to guide immunosuppression in the biomarker group, while the control group received standard care. Although graft function and AR incidence did not significantly differ between the groups, the biomarker-guided group underwent significantly fewer graft biopsies (12.5% vs. 47.4%, *p* = 0.027) and maintained lower tacrolimus levels without compromising safety (*p* = 0.006). These findings suggest that integrated omics monitoring can support more precise immunosuppressive management and reduce the need for invasive procedures in KTRs.

Papini et al. [14] reviewed the potential of metformin, a widely used antidiabetic drug, for repurposing in neurodegenerative diseases by exploring its impact on lysosomal-dependent mechanisms. Beyond its established role in glycemic control, metformin has been associated with protective effects against cardiovascular and neurodegenerative conditions, anti-tumor activity, and lifespan extension. The review highlights how metformin influences lysosomal targets and pathways—including endosomal Na^+^/H^+^ exchangers, presenilin enhancer 2 (PEN2), AMPK activation via the lysosomal pathway, and transcription factor EB (TFEB)—which are increasingly recognized as critical regulators in neurodegeneration. Lysosomal dysfunctions, particularly those affecting autophagy, acidification, and biogenesis, are emerging hallmarks of both inherited and sporadic neurodegenerative disorders. By modulating these pathways, metformin could help restore cellular homeostasis and support the clearance of protein aggregates. However, the authors emphasize that despite the promising therapeutic potential, many aspects remain to be clarified—such as the tissue-specific mechanisms and optimal dosing—before metformin can be reliably repositioned in the context of neurological diseases.

Ahamed et al. [15] provided a comprehensive overview of the emerging roles of glucose-6-phosphate dehydrogenase (G6PD), a key metabolic enzyme, in human cancers. As the first and rate-limiting enzyme of the pentose phosphate pathway (PPP), G6PD not only supports nucleotide synthesis via ribose-5-phosphate production but also contributes to cellular redox balance by generating NADPH. The review highlights how increased G6PD expression and PPP flux have been observed in various tumors and are associated with key cancer hallmarks such as enhanced proliferation, resistance to cell death, metabolic reprogramming, and metastatic potential. Targeting G6PD has been shown to suppress tumor growth, sensitize cells to chemotherapy, and reduce metastatic capacity, suggesting its therapeutic relevance. The authors discuss the development of small-molecule G6PD inhibitors, noting both their promise and the challenges that remain, such as potential side effects and specificity. Overall, G6PD emerges as a promising metabolic target in oncology, warranting further investigation for future cancer therapies.

Collectively, the contributions presented in this Special Issue reflect the multifaceted nature of translational medicine, showcasing how molecular insights can be leveraged to improve diagnostic precision, therapeutic efficacy, and patient outcomes. From novel biomarker discovery and risk stratification tools to the exploration of metabolic, inflammatory, and immune pathways, each study emphasizes the importance of an integrated, bench-to-bedside approach in tackling complex diseases. The integration of omics technologies, personalized treatment strategies, and innovative therapeutic targets demonstrate a clear shift toward a more precise, predictive, and preventive model of medicine. As translational research continues to evolve, it will be essential to strengthen interdisciplinary collaboration and ensure that scientific innovations are not only biologically sound but also clinically meaningful and accessible. By fostering dialogue between basic science and clinical practice, this collection aims to inspire continued innovation, ultimately advancing the shared goal of delivering more effective, equitable, and personalized care to patients worldwide.
